# Thoracoscopic Trans-mitral Septal Myectomy for Hypertrophic Obstructive Cardiomyopathy in the Elderly

**DOI:** 10.3389/fcvm.2022.827860

**Published:** 2022-03-09

**Authors:** Peijian Wei, Jian Liu, Jiexu Ma, Yanjun Liu, Tong Tan, Hongxiang Wu, Wei Zhu, Zhao Chen, Jimei Chen, Jian Zhuang, Huiming Guo

**Affiliations:** ^1^Department of Cardiovascular Surgery, Guangdong Cardiovascular Institute, Guangdong Provincial People's Hospital (Guangdong Academy of Medical Sciences), Guangzhou, China; ^2^Department of Adult Cardiac Ultrasound Medicine, Guangdong Cardiovascular Institute, Guangdong Provincial People's Hospital (Guangdong Academy of Medical Sciences), Guangzhou, China

**Keywords:** thoracoscopic, trans-mitral, septal myectomy, hypertrophic obstructive cardiomyopathy, elderly

## Abstract

**Background:**

The thoracoscopic trans-mitral approach can not only facilitate exposure of the ventricular septum, mitral valve, and subvalvular apparatus, it also enables the surgeons to perform concomitant mitral valve intervention. This study aimed to determine the safety and efficacy of thoracoscopic trans-mitral septal myectomy in elderly patients with hypertrophic obstructive cardiomyopathy (HOCM).

**Methods:**

We reviewed the demographic to clinical characteristics and outcomes of patients who underwent thoracoscopic trans-mitral septal myectomy in our center between April 2019 and April 2021. The population was divided into a younger group (<60 years) and an elderly group (≥60 years).

**Results:**

There were 46 and 20 patients in the younger and elderly groups, respectively. The majority of patients in the elderly group were female (39.1 vs. 80.0%, *P* < 0.01). Patients in the elderly group were more likely to be in New York Heart Association Class IV (2.2 vs. 80.0%, *P* < 0.01). The European System for Cardiac Operation Risk Evaluation II predicted mortality rates were significantly higher (3.97 ± 1.81 vs. 1.62 ± 0.86%, *P* < 0.01) in the elderly group. In the elderly group, a patient converted to median sternotomy due to left ventricular posterior free wall rupture following septal myectomy and mitral bioprosthetic valve replacement. The patient then underwent double-patch sandwich repair for rupture and mitral mechanical valve replacement and was eventually discharged. All patients in the elderly group were discharged, while one in the younger group died. No patient in the elderly group required permanent pacemaker implantation vs. one in the younger group. Patients in the elderly group were more likely to spend more time in the intensive care unit than those in the younger group (5.44 ± 5.80 days vs. 3.07 ± 2.72, *P* < 0.05). However, there was no significant intergroup difference in in-hospital mortality or complications. Importantly, the left ventricular outflow tract pressure gradient was significantly decreased from 96.15 ± 32.89 mmHg to 8.2 ± 3.42 mmHg with no residual obstruction in the elderly group. The interventricular septal thickness was significantly decreased from 19.73 ± 3.14 mm to 11.30 ± 2.23 mm. Postoperative mitral regurgitation severity was significantly improved in the elderly group.

**Conclusion:**

This study demonstrated that thoracoscopic trans-mitral septal myectomy is a feasible option for selected elderly patients with satisfactory outcomes similar to those of young patients.

## Introduction

Hypertrophic obstructive cardiomyopathy (HOCM) is among the most common inherited cardiovascular diseases with a reported prevalence of 1:200 to 1:500 in the United States (1). HOCM causes left ventricular outflow tract obstruction (LVOTO) at rest or with provocation in approximately 75% of patients (2). Septal reduction therapy, including septal myectomy (SM) and alcohol septal ablation (ASA) (3), is recommended (Class IB) for patients with severe symptomatic HOCM who are refractory to maximum medical therapy to relieve LVOTO (4). SM remains the gold standard surgical treatment for most drug-refractory symptomatic patients (5). However, despite continued debates on the effectiveness of SM and ASA (6), the latter is now the preferred treatment option for patients of advanced age due to the predicted high risk of morbidity and mortality. The advantages of ASA lead to its increased use vs. the reduced use of SM worldwide, especially in Europe (7). Therefore, several experienced surgeons successively appealed to more SM surgeons and high-volume centers (8, 9). Furthermore, there are limited data and reports in terms of surgical outcomes of the subgroup of HOCM patients older than 60 years. To our knowledge, there are no studies of the safety and effectiveness of thoracoscopic trans-mitral SM in elderly individuals. Our study aimed to investigate whether thoracoscopic trans-mitral SM is feasible in elderly vs. young populations.

## Materials and Methods

### Patient Population and Study Design

We retrospectively collected and analyzed the clinical data of consecutive HOCM patients (≥18 years) who underwent thoracoscopic trans-mitral SM in our center between April 2019 and April 2021. The final study population consisted of 66 consecutive patients with HOCM and was divided into two groups based on age: younger group (<60 years, 46 patients) and elderly group (≥60 years, 20 patients). The inclusion criteria were: (I) admission transthoracic echocardiography (TTE) confirmed a maximal end-diastolic left ventricular wall thickness ≥15 mm in any segment, excluding secondary to hypertensive heart disease or severe aortic stenosis; (II) left ventricular outflow tract peak gradient (LVOTPG) 50 mmHg (1 mmHg = 0.133 kPa) or greater at rest or with provocation such as the Valsalva maneuver; and (III) severe drug-refractory symptoms with New York Heart Association (NYHA) functional class II, III, and IV. The exclusion criteria were: (I) end-stage heart failure; (II) organic aortic valve diseases or coronary artery diseases requiring concomitant aortic valve replacement or coronary artery grafting; (III) malignancy; and (IV) major vascular diseases or malformations such as in the femoral arteries, iliac arteries, or abdominal aorta. The primary point was in-hospital death. Secondary points were primary adverse events such as permanent pacemaker implantation, iatrogenic septal defect, and low cardiac output syndrome.

### Preoperative Evaluation and Surgical Planning

All patients underwent preoperative transaortic echocardiography to (I) confirm atrioventricular valve; (II) identify mitral and papillary anomalies, including elongated leaflets and papillary muscle displacement; (III) identify systolic anterior motion (SAM) of the mitral valve; (IV) measure peak left ventricular outflow tract velocity by continuous-wave Doppler echocardiography and estimate LVOTPG using the Bernoulli equation (=4v^2^); and (V) measure end-diastolic left ventricular wall thickness in each segment from basal to apical using the British Society of Echocardiography practical guideline (10), especially basal anteroseptum, basal inferoseptum, mid anteroseptum, and mid inferoseptum. To further understand the extent and geometry of hypertrophic septum and plan surgical resection, preoperative cardiac magnetic resonance imaging, and cardiac computed tomography were performed in all patients. In some anatomically complex cases, surgeons performed myectomy simulations using patient-specific three-dimensional reconstruction and printing models. This was described in detail in our previous article (11).

### Surgical Techniques

**Figure 1:**
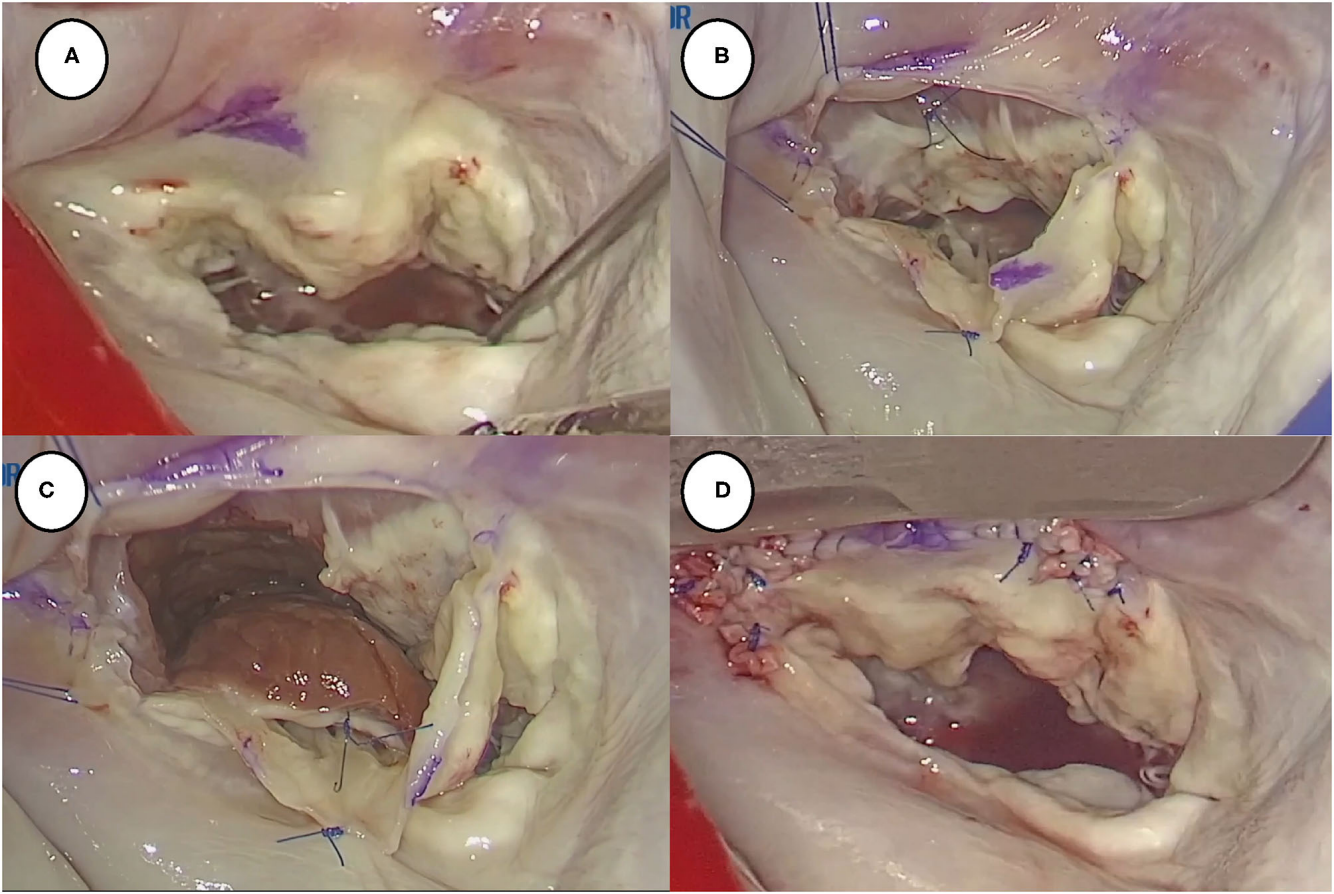
Summary of thoracoscopic trans-mitral septal myectomy. **(A)** Transatrial approach to expose mitral valve and subvalvular apparatus; **(B)** detached anterior mitral valve leaflet 1–2 mms away from annulus to further expose hypertrophic interventricular septum; **(C)** performed myectomy using sharp blade; **(D)** reattached anterior mitral valve leaflet to annulus.

Our operative techniques of thoracoscopic trans-mitral SM were described in detail in previous publications and are summarized here (11). Briefly, after the introduction of general anesthesia, routine transesophageal echocardiography was performed to further confirm the cardiac anatomy, valvular function, SAM, and hypertrophic septum characteristics. The mitral valve was exposed using a thoracoscopy under routine peripheral cardiopulmonary bypass and myocardial protection. After inspection and assessment of the mitral valve and subvalvular apparatus, the anterior mitral leaflet is detached a few millimeters away from mitral annulus from the commissure to the commissure to optimize visualization. Next, SM was performed about 3–5 mm to the right of the nadir of the right aortic sinus, leftward to the anterior commissure of the mitral valve, and downward to the apex. The actual resection scope and depth were based on preoperative evaluation and planning, surgical simulation, and individual anatomy. Avoiding the membranous septum is key to preventing postoperative atrioventricular block. Thickened secondary chordal, mitral leaflet, and papillary muscle anomalies were resected or released as appropriate. Next, the anterior mitral leaflet was reattached to the annulus using a continuous 5-0 Prolene suture after resection. The left ventricle was then liberally irrigated with saline to remove any residual debris and analyze the mitral valve anatomy and function. Mitral valve replacement or mitral valvuloplasty techniques, including edge-to-edge, triangular resection, ring annuloplasty, and artificial chordal implantation, were performed based on the water test results (Figure 1). The operation continued routinely: the left atriotomy was closed, and cardiopulmonary bypass was weaned. Transesophageal echocardiography was then performed immediately to confirm relief of the LVOTO, absence of a ventricular septal defect, and mitral regurgitation severity. Second aortic cross-clamping was required for redo procedures once the LVOTPG was ≥30 mmHg and/or moderate or severe mitral regurgitation was present. A ventricular temporary epicardial pacing electrode was routinely placed in all cases. In cases of developing postoperative complete atrioventricular block, permanent pacemaker would be implanted.

### Follow-Up

Data were obtained from departmental databases. All patients underwent a TTE assessment before hospital discharge and during follow-up. All patients were routinely followed-up at our cardiovascular surgery outpatient clinic or investigated by telephone. Clinical evaluation and TTE were performed at 30 days, 6 months, 1 year, and annually thereafter. None of the patients were lost to follow-up. The follow-up ended on August 31, 2021. The follow-up aimed to record any adverse events, including death, complete atrioventricular block, recurrent LVOTO, severe mitral regurgitation, stroke, and rehospitalization.

### Statistical Analysis

Descriptive statistics for categorical variables are reported as frequency and percentage and were compared using the chi-squared test or Fisher's exact test as appropriate. Continuous variables are presented as mean ± standard deviation and were compared using an independent samples *t*-test (normal distribution) or Mann–Whitney test (non-normal distribution) as appropriate. Two-sided *P* < 0.05 were considered statistically significant. The statistical analyses were performed using R (R x64 version 4.0.2, R Foundation for Statistical Computing, Vienna, Austria). The power analysis is normally run using the R package “pwr”. The calculated statistical power was 0.84 with an effect size of 0.8 and a significance level of 0.05.

## Results

### Study Population

From April 2019 to April 2021, 66 consecutive HOCM patients underwent thoracoscopic trans-mitral SM for relief of LVOTO and alleviation of symptoms. The baseline demographic and clinical characteristics of the two groups are shown in Table 1. Among them, 20 patients were older than 59 years with a mean age of 67.25 ± 4.98 years (range, 60–76 years) and 46 were younger than 60 years with a mean age of 47.30 ± 10.05 years (range, 18–59 years). We noted significant gender intergroup differences: females comprised 80% of the elderly group and 39.1% of the younger group. Patients in the elderly group were more likely to have severe symptoms (New York Heart Association functional class IV: 35 vs. 2.2%, *P* < 0.01). Symptoms such as syncope or amaurosis were present in 5 patients (25%) in the elderly group and 5 patients (10.9%) in the younger group. There was no significant intergroup difference in the rate of family history of hypertrophic cardiomyopathy; history of ASA; history of stroke; diabetes; coronary artery disease; renal dysfunction; or chronic obstructive pulmonary disease. Notably, the elderly group had a higher incidence of hypertension (60 vs. 26.1%, *P* < 0.01) and organic mitral regurgitation (25 vs. 6.5%, *P* < 0.05) than the younger group. The surgical mortality predicted by the European System for Cardiac Operative Risk Evaluation II was significantly higher in the elderly group (3.97 ± 1.81% vs. 1.62 ± 0.86%, *P* < 0.01). No significant intergroup differences were observed in terms of all echocardiographic paraments except for interventricular septum thickness (elderly group 21.37 ± 3.66 mm vs. younger group 19.23 ± 3.50 mm, *P* < 0.05). Patients with 20–30 mm and >30 mm interventricular septum thickness constituted 60.9% (*N* = 28) and 6.5% (*N* = 3) and 45% (*N* = 9) and 0% of the population of the young and elderly groups, respectively. In addition, SAM and moderate or severe mitral regurgitation were diagnosed in most patients of the population in both groups.

**Table 1:** Demographic and clinical characteristics.

	**Younger group (<60 years)**	**Elderly group (≥60 years)**	***p***
*n*	46	20	
Female	18 (39.1%)	16 (80%)	<0.01
Age	47.30 ± 10.05	67.25 ± 4.98	<0.01
BMI	24.21 ± 3.41	24.53 ± 2.70	0.72
NYHA classification (%)			<0.01
II	13 (28.3%)	3 (15%)	
III	32 (69.6%)	10 (50%)	
IV	1 (2.2%)	7 (35%)	
Syncope/Amaurosis	5 (10.9%)	5 (25%)	0.16
Family history of HCM	4 (8.7%)	1 (5%)	1
History of alcohol ablation	3 (6.5%)	0	0.55
Comorbidity	25 (54.3%)	13 (65%)	0.59
Hypertension	12 (26.1%)	12 (60%)	<0.01
Diabetes	8 (17.4%)	1 (5%)	0.26
Non-significant coronary artery disease^*^	2 (4.3%)	4 (20%)	0.06
Renal dysfunction	2 (4.3%)	0	1
Chronic obstructive pulmonary disease	4 (8.7%)	1 (5%)	1
History of stroke	2 (4.3%)	0	1
Smoke	9 (19.6%)	0	<0.05
EuroSCORE II predicted mortality	1.62 ± 0.86%	3.97 ± 1.81%	<0.01
MR (%)			0.06
No	2 (4.3%)	0	
Mild	9 (19.6%)	1 (5%)	
Moderate	19 (41.3%)	5 (25%)	
Severe	16 (34.8%)	14 (70%)	
Organic MR	3 (6.5%)	5 (25%)	<0.05
SAM	42 (91.3%)	18 (90%)	1
IVS thickness (mm)	21.37 ± 3.66	19.23 ± 3.50	<0.05
IVS Class			0.18
<20 mm	15 (32.6%)	11 (55%)	
20–30 mm	28 (60.9%)	9 (45%)	
>30 mm	3 (6.5%)	0	
EF (mm)	66.07 ± 10.80	67.50 ± 4.49	0.57
LAD (mm)	44.87 ± 7.25	43.90 ± 5.68	0.60
LVDd (mm)	42.67 ± 4.26	43.50 ± 5.53	0.51
LVDs (mm)	26.33 ± 3.64	26.70 ± 4.04	0.71
LVOTPG (mmHg)	81.41 ± 34.78	96.15 ± 32.89	0.113

*BMI, body mass index; NYHA, New York Heart Association; EF, ejection fraction; EuroSCORE II, European System for Cardiac Operative Risk Evaluation II; HCM, hypertrophic cardiomyopathy; IVS, interventricular septum; LAD, left atrial dimension; LVDd, left ventricular end-diastolic dimension; LVDs, left ventricular end-systolic dimension; LVOTPG, left ventricular outflow tract peak gradient; MR, mitral regurgitation; SAM, systolic anterior motion of the mitral leaflet*.

* *These patients didn't meet the indications for coronary artery bypass grafting or percutaneous coronary intervention*.

### Operative Details and Clinical Outcomes

Table 2 summarizes the operative details and clinical outcomes of both groups. Operation duration, cardiopulmonary time, and aortic cross-clamping time were similar between the groups. One patient converted intraoperatively to a median sternotomy due to an iatrogenic ventricular septal defect in the younger group; the defect was subsequently closed using the double-patch sandwich technique. The patient underwent the insertion of an intra-aortic balloon pump due to postoperative low cardiac output syndrome and eventually recovered well. In addition, a left ventricular posterior free wall rupture occurred in one patient who underwent SM and mitral bioprosthetic valve replacement in the elderly group. The patient then underwent double-patch sandwich repair for the rupture and mitral mechanical valve replacement. Finally, the patient was discharged home smoothly despite the development of low cardiac output syndrome. The proportions of patients who underwent mitral valve replacement in the elderly and the younger groups were 45% (*n* = 9) and 23.9% (*n* = 11), respectively. Given the inclusion and exclusion criteria, no significant intergroup differences were observed in concomitant procedures. Second aortic cross-clamping was recorded in five patients in the younger group vs. two patients in the elderly group. Second aortic cross-clamping mainly occurred in the early cases due to the significant residual mitral regurgitation resulting from leaflet tear at suture, especially at commissures. In the later cases, we reinforced the continuous suture with felts (see Figure 1D). In addition, patients in the younger group were more likely to undergo mitral valve repair than those in the elderly group (76.1 vs. 55%). Importantly, patients in the younger group had shorter ventilation time than those in the elderly group, which led to a significantly shorter intensive care unit stay. The length of hospitalization seemed longer in patients of the elderly group (35.95 ± 20.95 days vs. 27.72 ± 13.44 days, *P* = 0.06). It may relate to the preoperative frail status and poor ability of recovery of the elderly population. Notably, the elderly group had a higher incidence of hypertension (60 vs. 26.1%, *P* < 0.01) and non-significant coronary artery disease (20 vs. 4.3%, *P* = 0.06) than the younger group. The incidence of postoperative complications was low in both groups. There were no intergroup differences in the rates of postoperative blood transfusion, reintubation, reoperation for bleeding, and in-hospital mortality. There were no deaths in the elderly group throughout the study period; however, one patient died of low cardiac output syndrome on postoperative day 2 in the younger group. In addition, 18 of 46 patients (39.1%) in the younger group vs. 7 of 20 patients (35%) in the elderly group developed new-onset left bundle branch block. Of note, one patient developed a complete atrioventricular block that required permanent pacemaker implantation.

**Table 2:** Operative characteristics and outcomes.

	**Younger group (<60 years)**	**Elderly group (≥60 years)**	***p***
*n*	46	20	
Operation time (mins)	284.93 ± 71.70	315.25 ± 135.57	0.24
Cardiopulmonary bypass (mins)	196.20 ± 63.14	208.50 ± 91.86	0.53
Aortic cross-clamping (mins)	129.09 ± 42.01	125.30 ± 46.97	0.75
Conversion to median sternotomy	1 (2.2%)	1 (5%)	1
Mitral intervention			0.14
Anterior mitral leaflet (AML) extension	19 (41.3%)	4 (20%)	
AML directly reattached	16 (34.8%)	7 (35%)	
Mitral valve replacement	11 (23.9%)	9 (45%)	
Concomitant procedures			0.35
Atrial septal defect repair	9 (19.6%)	1 (5%)	
Left Atrial Appendage Closure (LAAC)	1 (2.2%)	1 (5%)	
Tricuspid Valve Repair (TVP)	1 (2.2%)	0	
Maze IV+ LAAC	3 (6.5%)	0	
Maze IV+ LAAC+TVP	1 (2.2%)	1 (5%)	
Second aortic cross-clamping	5 (10.9%)	2 (10%)	1
Ventilation time (hours)	27.81 ± 42.64	38.72 ± 55.10	0.38
ICU stay (days)	3.07 ± 2.72	5.44 ± 5.80	<0.05
Blood transfusion	15 (32.6%)	5 (25%)	0.77
Reintubation	3 (6.5%)	1 (5%)	1
In-hospital mortality	1 (2.2%)	0	1
Complication	4 (8.7%)	2 (10%)	1
Low cardiac output syndrome	2 (4.3%)	1 (5%)	1
Reoperation for bleeding	1 (2.2%)	0	1
Rehospitalization	2 (4.3%)	1 (5%)	1
Length of Hospitalization (days)	27.72 ± 13.44	35.95 ± 20.95	0.06
Residual obstruction (>30 mmHg)	2 (4.3%)	0	1
New on-set (%)			0.91
Left anterior fascicular block (LAFB)	1 (2.2%)	0	
Left bundle branch block (LBBB)	18 (39.1%)	7 (35%)	
Right bundle branch block (RBBB)	1 (2.2%)	0	
Permanent pacemaker implantation	1 (2.2%)	0	
Atrial fibrillation	1 (2.2%)	0	

### Hemodynamic Outcomes

**Figure 2:**
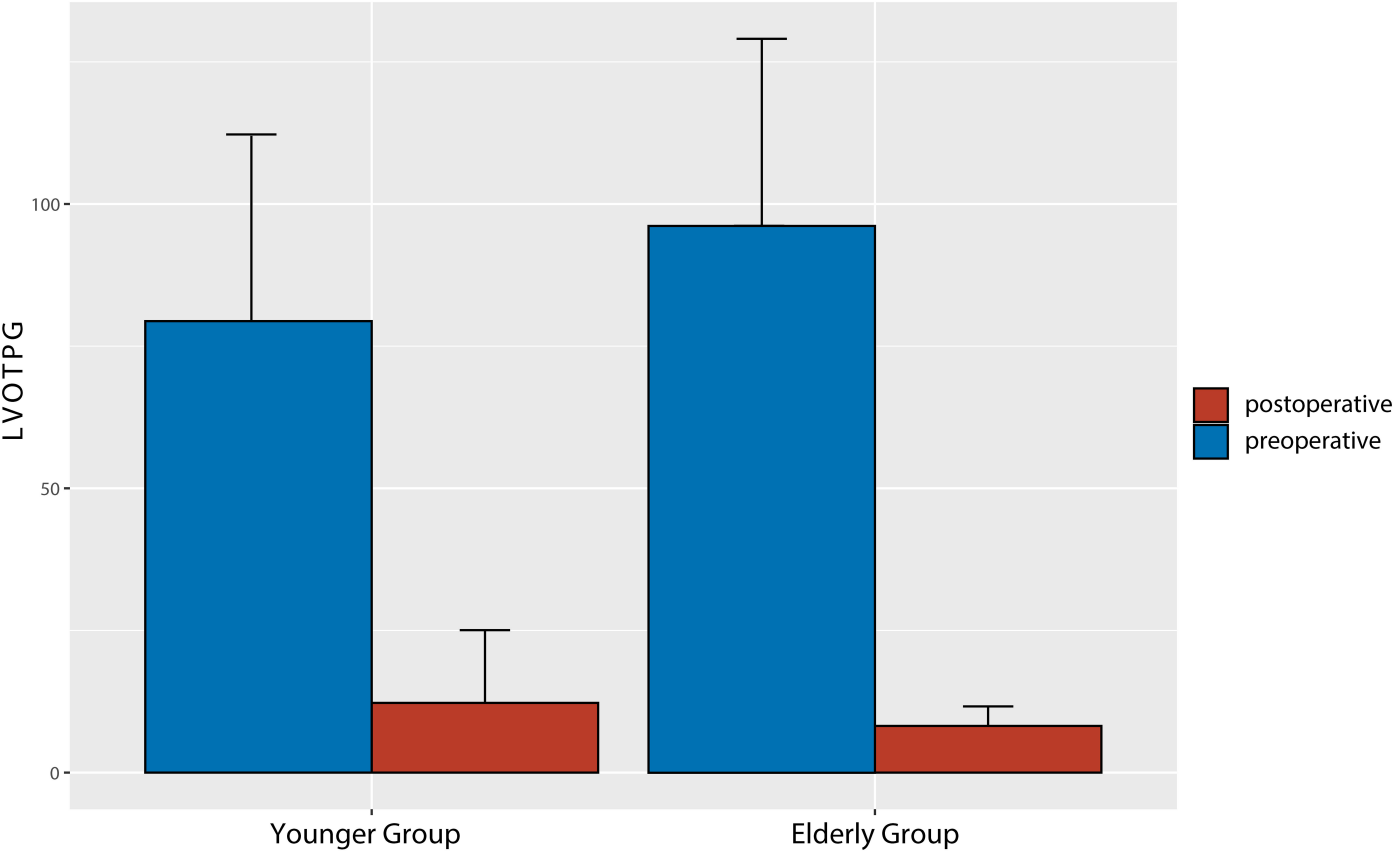
Comparison of pre- and post-operative left ventricular outflow tract pressure gradients of the two groups.

**Figure 3:**
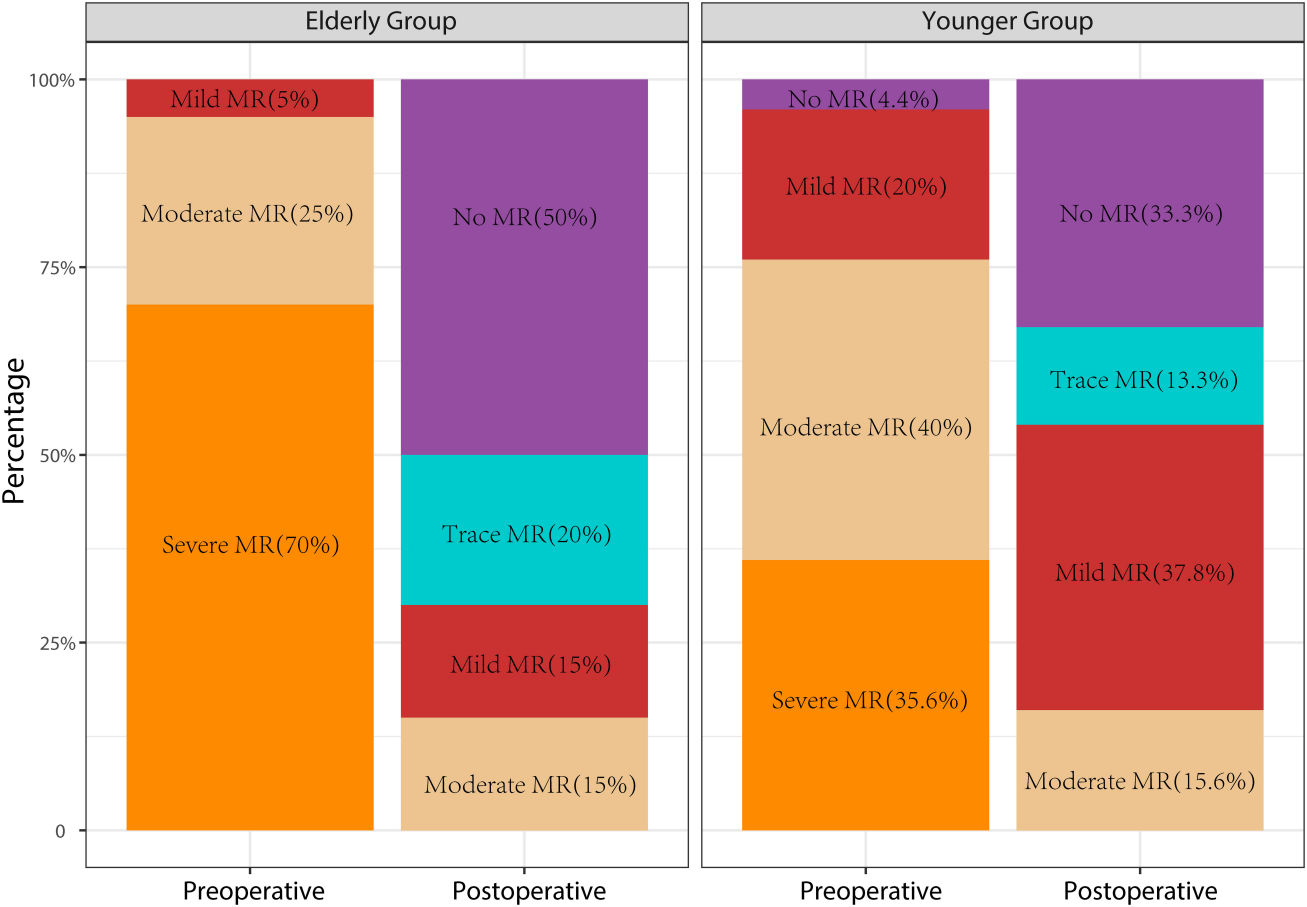
Evolution of pre- and post-operative mitral regurgitation severities of the two groups.

Perioperative TTE data are shown in Table 3. In the elderly group, LVOTO was completely relieved in all patients with a significantly decreased postoperative LVOTPG (8.20 ± 3.43 mmHg vs. 96.15 ± 32.89 mmHg, *P* < 0.01) and interventricular septum thickness (11.30 ± 2.23 mm vs. 19.23 ± 3.50 mm, *P* < 0.01) at discharge (Figure 2). In the younger group, LVOTPG decreased significantly from 81.41 ± 34.78 mmHg to 12.27 ± 12.79 mmHg (*P* < 0.01) and interventricular septum thickness decreased significantly from 21.37 ± 3.66 mm to 12.27 ± 12.79 mm on pre-discharge echocardiography. However, residual asymptomatic LVOTO was detected in two patients in the younger group. The SAM phenomenon disappeared in all patients in both groups. Preoperative mitral regurgitation severity significantly improved after the procedure in the elderly group (Figure 3). Most patients had no greater than mild postoperative mitral regurgitation, while moderate mitral regurgitation at discharge affected 15% (*n* = 3) of the elderly group and 15.6% (*n* = 7) of the younger group. Significant reductions in ejection fraction and left atrial dimension were observed in both groups. This indicates decreased left ventricular systolic function due to interventricular septal myocardium resection. In contrast, there was a significant increase in the left ventricular end-systolic dimension in both groups, indicating an increased left ventricular cavity volume.

**Table 3:** Transthoracic echocardiographic paraments.

	**Elderly group**			**Younger group**		
	**Preoperative**	**Postoperative**	***P*** **-value**	**Preoperative**	**Postoperative**	***P*** **-value**
*N*	20	20		45	45	
MR (%)			<0.01			<0.01
No	0	10 (50%)		2 (4.4%)	15 (33.3%)	
Trace	0	4 (20%)		0	6 (13.3%)	
Mild	1 (5%)	3 (15%)		9 (20%)	17 (37.8%)	
Moderate	5 (25%)	3 (15%)		18 (40%)	7 (15.6%)	
Severe	14 (70%)	0		16 (35.6%)	0	
SAM	18 (90%)	0	<0.01	41 (91.1%)	0	<0.01
EF	67.50 ± 4.49	64.20 ± 4.67	<0.05	67.43 ± 4.21	63.56 ± 4.88	<0.01
LAD (mm)	43.90 ± 5.68	40.20 ± 50	<0.05	44.87 ± 7.25	40.13 ± 5.86	<0.01
LVDd (mm)	43.50 ± 5.53	42.25 ± 6.87	0.53	42.82 ± 4.19	45.29 ± 4.34	<0.01
LVDs (mm)	26.70 ± 44	29.60 ± 4.79	<0.05	26.44 ± 3.59	29.89 ± 47	<0.01
IVS thickness (mm)	19.23 ± 3.50	11.30 ± 2.23	<0.01	21.37 ± 3.66	11.43 ± 3.14	<0.01
LVOTPG (mmHg)	96.15 ± 32.89	8.20 ± 3.43	<0.01	81.41 ± 34.78	12.27 ± 12.79	<0.01

*EF, ejection fraction; IVS, interventricular septum; LAD, left atrial dimension; LVDd, left ventricular end-diastolic dimension; LVDs, left ventricular end-systolic dimension; LVOTPG, left ventricular outflow tract pressure gradient; MR, mitral regurgitation; SAM, systolic anterior motion of the mitral leaflet*.

### Follow-Up Results

Patient follow-up was complete and averaged 14.11 ± 8.14 months in the elderly group and 13.05 ± 8.77 months in the younger group. None of the patients in the elderly group required reoperation for recurrent LVOTO and/or severe mitral regurgitation throughout the follow-up period, while redo mitral valve repair was performed on a patient from the younger group during follow-up. The patient was diagnosed with severe mitral regurgitation due to a tear of the reattached A1 segment. At the latest follow-up, all patients in the elderly group were classified as NYHA functional class I or II.

## Discussion

According to the 2020 American Heart Association/American College of Cardiology guidelines for the diagnosis and treatment of patients with hypertrophic cardiomyopathy (4), the mortality of SM is <1% in high-volume hypertrophic cardiomyopathy centers. SM is widely considered the gold treatment for HOCM patients. However, in the early 1990s, ASA emerged as an attractive alternative to surgery, particularly in patients of advanced age or those who have declined surgery (12). Considering that ASA has the dominant advantage of being less invasive with a shorter recovery period compared with SM, elderly patients prefer ASA over SM in the real world. Therefore, in most existing reports, the mean age of the study population was less than 60 years (13, 14). The clinical outcomes of SM in the elderly population have seldom been reported.

This subgroup recently attracted the attention of several researchers. In 2021, a retrospective analysis of 35 patients (>65 years) who underwent transaortic SM *via* median sternotomy by Wong et al. (15) demonstrated that SM is feasible for appropriately selected older HOCM patients with outcomes similar to those of younger patients. Apart from similar mortality rates, older patients were more likely to undergo concomitant coronary artery bypass. Pruna-Guillen et al. (16) showed that SM is an effective and safe procedure for elderly patients despite the need for concomitant procedures. In this series, the in-hospital mortality rate was 1.9% (*n* = 1) and 84.6% of the enrolled patients underwent concomitant procedures, including aortic valve replacement, coronary surgery, and Maze procedure. These two studies proved the encouraging outcomes of transaortic SM through traditional median sternotomy in elderly patients; however, to our knowledge, there are no reports on the clinical outcomes of thoracoscopic trans-mitral SM in this subgroup.

Indeed, transaortic SM first described in detail by Morrow et al. (17) is perceived as a classic procedure for treating HOCM. In past decades, the procedure evolved into extended myectomy and/or mitral valve interventions including plication of the elongated anterior mitral leaflet, resection of the secondary chordae, and papillary muscle reorientation (14). Although the transaortic approach is still the most commonly applied, it is not preferred for patients with a long segmental hypertrophic septum and various mitral and papillary anomalies due to suboptimal exposure. Several studies (18, 19) have reported that minimally invasive trans-mitral SM is feasible with satisfactory outcomes for patients with HOCM. Since the trans-mitral approach can provide better visualization of the hypertrophic septum and mitral subvalvular apparatus and perform mitral intervention simultaneously, Wehman et al. (20) recommended the trans-mitral approach as preferred for the surgical treatment of HOCM.

Here, we reported the clinical characteristics and outcomes of elderly patients who underwent thoracoscopic trans-mitral SM. A distinct gender-based intergroup difference was observed: females comprised 80% of the elderly group and 39.1% of the younger group. The elderly group was more likely to have severe symptoms (NYHA functional class IV: 35 vs. 2.2%, *P* < 0.01). These results were in part similar to the findings of Meghji et al. (21) that female patients were older and more symptomatic than male patients at the time of surgery. Their study suggested that there was no gender-based difference in survival after SM. Interestingly, patients in the younger group were more likely to smoke. This may correlate with the gender-based intergroup differences. Of note, the elderly group had a higher incidence of hypertension (60 vs. 26.1%, *P* < 0.01) and organic mitral regurgitation (25 vs. 6.5%, *P* < 0.05) than the younger group. Alashi et al. (3) reported that age was associated with worse long-term primary events (hazard ratio, 1.75; *P* < 0.01) in the subgroup of 597 HOCM patients. Similarly, the preoperative predicted risk of mortality by the European System for Cardiac Operation Risk Evaluation II was significantly higher in the elderly population than that in the young population (3.97 ± 1.81% vs. 1.62 ± 0.86%, *P* < 0.01). However, in our study, there were no deaths in the elderly group throughout the study period. There were no intergroup differences in the rates of postoperative blood transfusion, reintubation, reoperation for bleeding, in-hospital mortality, and the incidence of other postoperative complications. In the elderly group, LVOTPG decreased significantly from 96.15 ± 32.89 mmHg to 8.20 ± 3.43 mmHg and interventricular septum thickness decreased significantly from 19.23 ± 3.50 mm to 11.30 ± 2.23 mm before discharge. In addition, LVOTO was completely relieved and the SAM phenomenon disappeared in all patients. Our findings demonstrate that the thoracoscopic trans-mitral approach is a feasible and minimally invasive option for elderly patients that does not comprise quality.

### Study Limitation

This single-institution retrospective observational study was subject to the limitations inherent to a non-randomized and retrospective analysis. This study excluded patients with contraindications to thoracoscopic cardiac surgery, including major vascular diseases, organic aortic valve diseases, and severe coronary artery diseases. The prevalence of cardiovascular disease is usually higher in the elderly population. Another limitation of this study was the relatively short average follow-up time. The long-term outcomes of thoracoscopic trans-mitral SM in elderly patients require further investigation.

## Conclusion

The findings of the present study suggest that thoracoscopic trans-mitral septal myectomy is a feasible therapeutic option for selected elderly patients with HOCM that features satisfactory outcomes similar to those of the young population. However, long-term outcomes require further follow-up and investigation.

## Data Availability Statement

The original contributions presented in the study are included in the article/Supplementary Material, further inquiries can be directed to the corresponding author/s.

## Ethics Statement

The studies involving human participants were reviewed and approved by the Institutional Review Board of Guangdong Provincial People's Hospital. Written informed consent for participation was not required for this study in accordance with the national legislation and the institutional requirements.

## Author Contributions

PW and JL: conception, study design, data analysis and interpretation and drafting of manuscript. JM, YL, TT, HW, WZ, and ZC: collection and assembly of data. All authors contributed in manuscript writing and final approval of manuscript. All authors contributed to the article and approved the submitted version.

## Funding

This study was funded by Science and Technology Planning Project of Guangdong Province (2019B020230003 and 2018B090944002), Cardiovascular Special Project of Guangdong Provincial People's Hospital (2020XXG010), and Guangdong Special Funds for Science and Technology Innovation Strategy, China (Stability Support for Scientific Research Institutions Affiliated to Guangdong Province-GDCI 2021).

## Conflict of Interest

The authors declare that the research was conducted in the absence of any commercial or financial relationships that could be construed as a potential conflict of interest.

## Publisher's Note

All claims expressed in this article are solely those of the authors and do not necessarily represent those of their affiliated organizations, or those of the publisher, the editors and the reviewers. Any product that may be evaluated in this article, or claim that may be made by its manufacturer, is not guaranteed or endorsed by the publisher.
